# Nonsense-mediated mRNA decay efficiency varies in choroideremia providing a target to boost small molecule therapeutics

**DOI:** 10.1093/hmg/ddz028

**Published:** 2019-01-23

**Authors:** Hajrah Sarkar, Andreas Mitsios, Matthew Smart, Jane Skinner, Ailsa A Welch, Vasiliki Kalatzis, Peter J Coffey, Adam M Dubis, Andrew R Webster, Mariya Moosajee

**Affiliations:** 1 Development, Ageing and Disease, UCL Institute of Ophthalmology, London, UK; 2 Department of Genetics, Moorfields Eye Hospital NHS Foundation Trust, London, UK; 3 Department of Public Health & Primary Care, Norwich Medical School, University of East Anglia, Norwich, UK; 4 Inserm U1051, Institute for Neurosciences of Montpellier, Montpellier, Montpellier Cedex, France; 5 Department of Ophthalmology, Great Ormond Street Hospital for Children NHS Foundation Trust, London, UK

## Abstract

Choroideremia (CHM) is an x-linked recessive chorioretinal dystrophy, with 30% caused by nonsense mutations in the *CHM* gene resulting in an in-frame premature termination codon (PTC). Nonsense-mediated mRNA decay (NMD) is the cell’s natural surveillance mechanism that detects and destroys PTC-containing transcripts, with UPF1 being the central NMD modulator. NMD efficiency can be variable amongst individuals with some transcripts escaping destruction, leading to the production of a truncated non-functional or partially functional protein. Nonsense suppression drugs, such as ataluren, target these transcripts and read-through the PTC, leading to the production of a full length functional protein. Patients with higher transcript levels are considered to respond better to these drugs, as more substrate is available for read-through. Using Quantitative reverse transcription PCR (RT-qPCR), we show that *CHM* mRNA expression in blood from nonsense mutation CHM patients is 2.8-fold lower than controls, and varies widely amongst patients, with 40% variation between those carrying the same UGA mutation [c.715 C>T; p.(R239^*^)]. These results indicate that although NMD machinery is at work, efficiency is highly variable and not wholly dependent on mutation position. No significant difference in *CHM* mRNA levels was seen between two patients’ fibroblasts and their induced pluripotent stem cell-derived retinal pigment epithelium. There was no correlation between *CHM* mRNA expression and genotype, phenotype or *UPF1* transcript levels. NMD inhibition with caffeine was shown to restore *CHM* mRNA transcripts to near wild-type levels. Baseline mRNA levels may provide a prognostic indicator for response to nonsense suppression therapy, and caffeine may be a useful adjunct to enhance treatment efficacy where indicated.

## Introduction

Choroideremia (CHM; MIM: 303100) is an x-linked recessive chorioretinal dystrophy that affects approximately 1 in 50 000–100 000 individuals (1). CHM is characterized by a progressive loss of vision, starting with night blindness in early childhood, followed by peripheral field loss and eventually leading to complete blindness in late middle age. CHM is caused by mutations in the *CHM* gene (MIM: 300390), located on chromosome *Xq21.2*, it spans ~150 kb and is composed of 15 exons. It encodes the ubiquitously expressed 653 amino acid protein, Rab Escort Protein 1 (REP1). REP1 is involved in intracellular trafficking of vesicles and post-translational modification of Rab-proteins.

**Figure 1:**
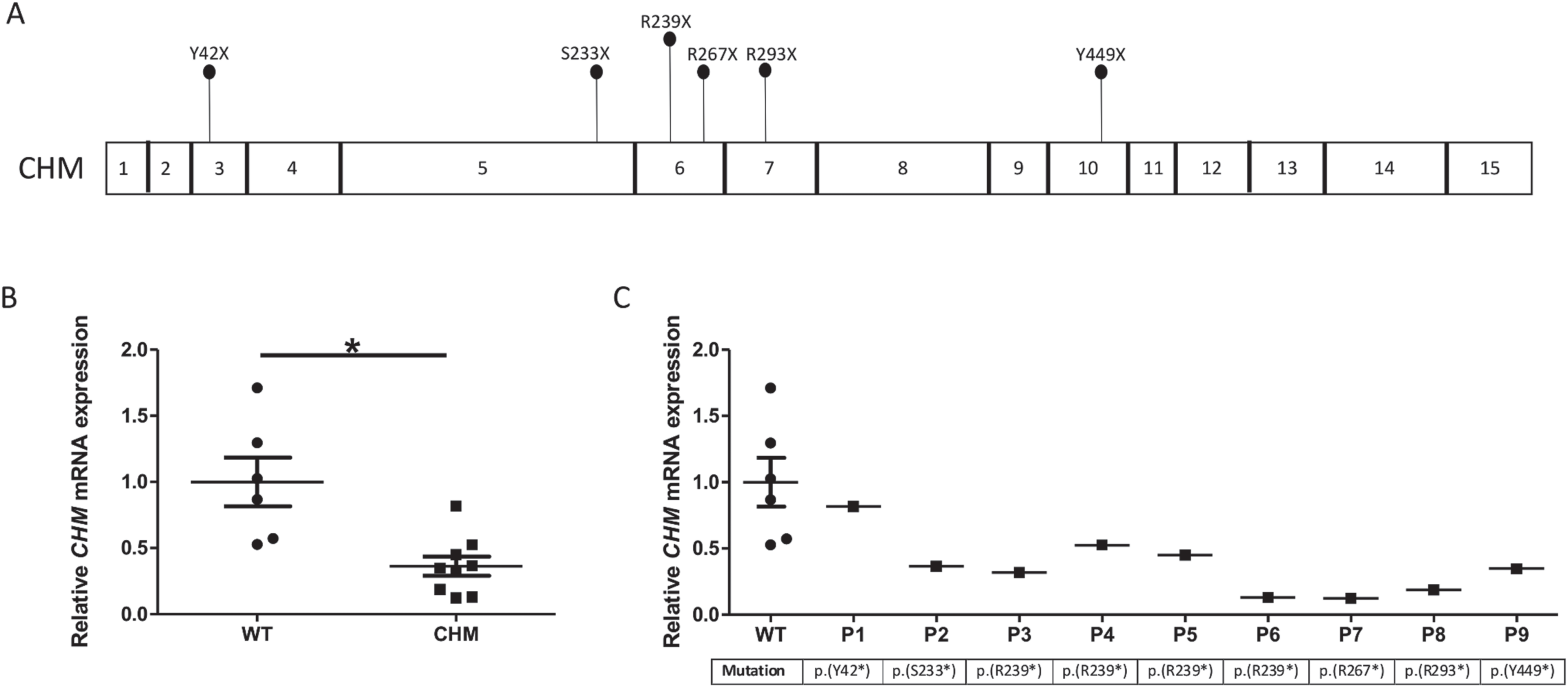
*CHM* mRNA expression is significantly reduced in patients. **(A)** Schematic of the *CHM* gene. Patient mutations used in this study are labelled. **(B)** Relative *CHM* mRNA expression in patients analysed by RT-qPCR. Patients have a 2.8-fold lower expression compared to control (^*^P = 0.008). **(C)** Relative CHM mRNA expression in patients, ordered by mutation position. No correlation was found between *CHM* mRNA expression and genotype. Data expressed as mean ± SEM.

Thirty percent of CHM cases are caused by nonsense mutations, resulting in an in-frame premature termination codon (PTC) (1). Nonsense-mediated mRNA decay (NMD) is the cell’s natural surveillance mechanism, which detects and destroys PTC-containing transcripts. Typically, PTCs found more than 50–55 nucleotides upstream of the last exon–exon junction are described as being marked for destruction (2). However, exceptions to this rule have been observed. For example, in T-cell receptor-β, PTCs located within 50 nucleotides of the last exon–exon junction are still degraded (3). In the case of the β-globin gene (MIM: 141900), PTCs in close proximity to an AUG codon evade NMD and trigger translation re-initiation (4). NMD is a complex multifactorial mechanism that is intrinsically linked to translation. UPF1 is the central NMD factor; it is an RNA dependent ATPase and an ATP-dependent RNA helicase that is recruited to mRNA and undergoes a cycle of phosphorylations and dephosphorylations (5). Although NMD is described primarily as a surveillance mechanism, it also plays an important role in the regulation of normal gene expression and response to cellular stress (5,6).

**Table 1:** CHM male affected patients enrolled in this study

Patient	Age	cDNA change	Amino acid change	Stop introduced	Exon	FAF area (mm^2^)
P1	28	c.126 C>G	p.(Y42^*^)	UAG	3	1.77
P2	50	c.698 C>G	p.(S233^*^)	UGA	5	0.41
P3	28	c.715 C>T	p.(R239^*^)	UGA	6	22.32
P4	50	c.715 C>T	p.(R239^*^)	UGA	6	19.71
P5	62	c.715 C>T	p.(R239^*^)	UGA	6	19.62
P6	72	c.715 C>T	p.(R239^*^)	UGA	6	2.66
P7	49	c.799 C>T	p.(R267^*^)	UGA	6	18.55
P8	43	c.877 C>T	p.(R293^*^)	UGA	7	10.98
P9	58	c.1347 C>G	p.(Y449^*^)	UAG	10	6.27

FAF analysed using the Heidelberg area tool, Heidelberg Engineering.

Variants correspond to RefSeq NM_000390.4.

**Figure 2:**
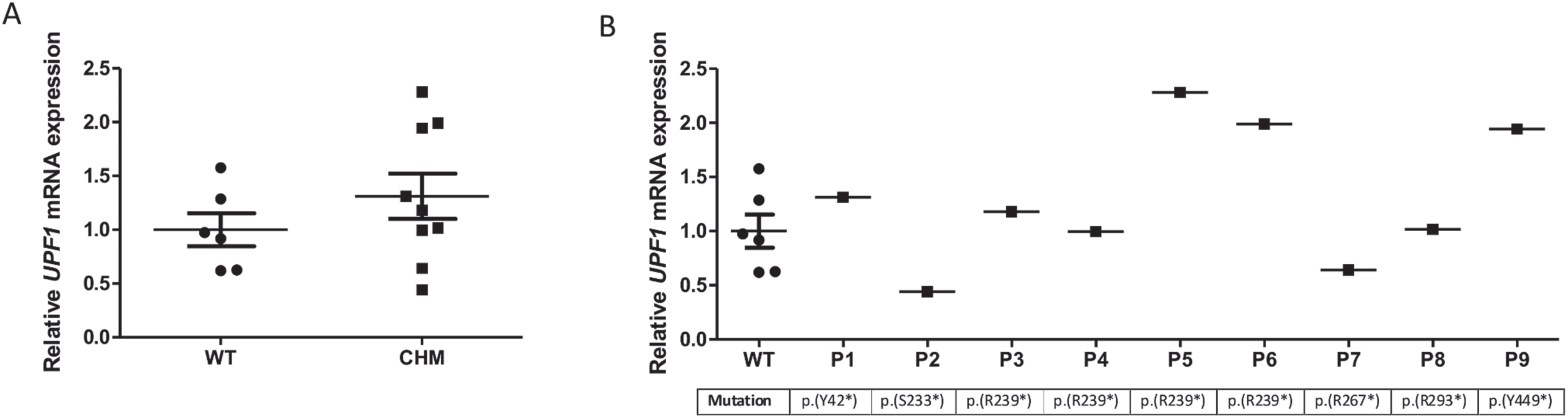
*UPF1* mRNA expression is widely variable amongst patients. **(A)** Relative *UPF1* mRNA expression in patients analysed by RT-qPCR. No significant difference was found between patients and controls. **(B)** Relative *UPF1* mRNA expression in patients, ordered by mutation position. No correlation was found between *UPF1* mRNA expression and genotype. Data expressed as mean ± SEM.

Some PTC-containing transcripts escape NMD, leading to the expression of a truncated partially functional or non-functional protein. Nonsense suppression drugs exploit these PTC-containing transcripts. They bind to the ribosomal subunit and increase the ability of a near cognate aminoacyl-tRNA to compete with the eukaryotic release factors for binding to the A-site. An amino acid is added to the growing polypeptide chain, effectively allowing ‘read-through’ of the PTC, leading to production of the full length functional protein (7). We have previously shown that small molecule drugs, PTC124 and PTC414, restore rep1/REP1 activity in the *chm^ru848^* zebrafish model and a patient *CHM^Y42X^* fibroblast cell line (8), whereas it was less effective in *CHM^K248X^* fibroblasts and induced pluripotent stem cell (iPSC)-derived retinal pigment epithelium (RPE; 9).

It has been suggested that the response to nonsense suppression drugs is greater in patients with higher baseline transcripts, providing more substrate for drug action, as a result of lower NMD efficiency (10). NMD efficiency is known to be variable between individuals (11); however, it is not yet fully understood what governs these differences. Linde *et al.* (10) found patients with the same mutation, p.(W1282^*^), in the cystic fibrosis transmembrane conductance regulator gene (*CFTR*; MIM: 602421), had widely variable transcript levels, indicating that NMD is not entirely governed by PTC position alone.

Baseline mRNA levels may be used as prognostic indicators of treatment outcome and inhibition of the NMD pathway could be used as an adjunct to boost transcripts for nonsense suppression. Caffeine has been identified as an NMD inhibitor, due to its inhibitory action on SMG1 kinase activity (12). Ullrich’s disease (MIM: 254090) is a muscular dystrophy, caused by mutations in the collagen VI genes. Caffeine has been shown to rescue the phenotype in Ullrich’s disease fibroblasts, by increasing the level of collagen VI α2 mRNA and protein, resulting in efficient integration into the collagen VI triple helix (13). Co-administration of the NMD inhibitor NMDI-1 with the nonsense suppression drug, gentamicin, has been shown to restore full length protein in a model of Hurler syndrome (MIM: 607014; 14).

In preparation for a clinical trial with PTC124 (ataluren) for CHM, we examined NMD efficiency in nonsense mutation CHM patients, determining relative *CHM* and *UPF1* mRNA transcript levels in blood, fibroblasts and iPSC-derived RPE. We have shown that NMD efficiency is variable in nonsense mutation CHM patients and does not correlate with genotype or phenotype. NMD inhibition increases *CHM* transcript levels and could be explored as an adjunct for the treatment of nonsense-mediated diseases.

## Results

### Variable *CHM* mRNA expression in patient whole blood

*CHM* transcript levels in whole blood from nine CHM male patients with nonsense mutations (mean age 49 ± 15 years) and six age- and sex-matched healthy controls (mean age 45 ± 15 years) were measured using Quantitative reverse transcription PCR (RT-qPCR). Patient mutations are shown in Figure 1A and Table 1. *CHM* transcript levels were reduced in all patients. Overall, mean *CHM* mRNA expression in patients was significantly reduced to 36.3 ± 7.3% of control (*P* = 0.002; Fig. 1B). A large variability in transcript levels amongst patients was seen, ranging from 12.5% to 81.2% of wild-type levels. These results indicate that a proportion of transcripts are escaping NMD. In our cohort, four patients have a c.715 C>T; p.(R239^*^) UGA mutation; in these patients, *CHM* transcript levels ranged from 13% to 52.6%. No correlation between *CHM* mRNA transcript level and genotype was found (Fig. 1C).

We next analysed the levels of *UPF1* in patient blood to determine if there was a correlation between expression of genes encoding proteins involved in the NMD pathway and mRNA levels of *CHM*. There was no significant difference in *UPF1* expression between patients and controls. However, there was a large variation in *UPF1* mRNA expression amongst patients, ranging from 44.3% to 228.1%, compared to wild-type levels (Fig. 2). Except for P2 and P7, all other patients had higher *UPF1* expression compared to controls. No correlation between *CHM* and *UPF1* transcript levels was observed (*r* = 0.07).

A genotype–phenotype correlation does not exist for CHM patients (15). In this population, the relationship between phenotype [age and fundus autofluorescence (FAF) size] and *CHM* transcript levels was investigated, but no statistically significant correlation was found (*P* = 0.21). Although it is important to note that in this population, there was also no correlation between age and FAF area (*P* = 0.53). So while the multivariate linear model did not suggest statistical significance, it did improve the correlation over any single factor correlation. Therefore, further investigation in a larger patient cohort may be needed to determine actual interaction.

### Tissue-specific NMD variation

**Figure 3:**
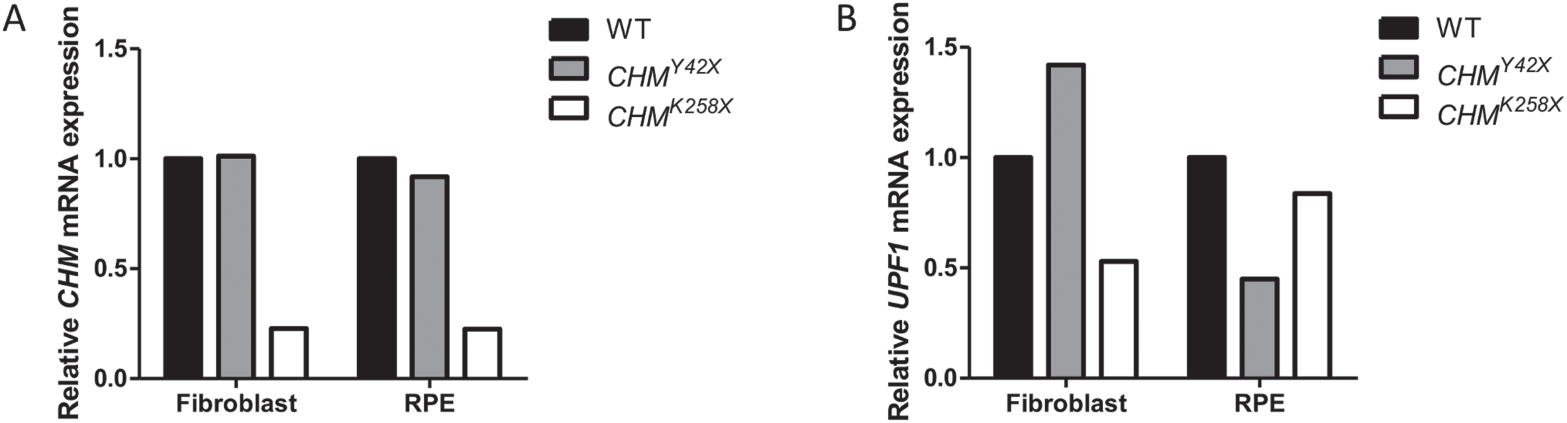
No variation in NMD efficiency was found between cell types. Relative **(A)***CHM* and **(B)***UPF1* mRNA expression in *CHM^Y42X^* (grey) and *CHM^K258X^* (white) fibroblasts and iPSC-derived RPE.

Previous studies have shown that NMD efficiency varies between different murine tissues (16). In order to investigate tissue-specific NMD variation, we have analysed *CHM* and *UPF1* expression in fibroblasts and iPSC-derived RPE from two unrelated patients (i) p.(Y42^*^) and (ii) p.(K258^*^). *CHM* transcript levels in fibroblasts and iPSC-derived RPE for p.(Y42^*^) were 101 and 92% relative to an age-matched healthy control and for p.(K258^*^) were 22.8% and 22.6%, respectively (Fig. 3A). *UPF1* transcript levels for p.(Y42^*^) were 142% and 45% and for p.(K258^*^) were 52.9% and 83.7%, respectively (Fig. 3B). Our results show that the *CHM* transcript levels in both cell types are similar for each patient; however, *UPF1* expression varied considerably amongst the different tissues. *CHM* mRNA transcripts in p.(Y42^*^) are present at wild-type levels, indicating that this transcript is escaping NMD.

### NMD inhibition increases *CHM* mRNA expression in fibroblasts

**Figure 4:**
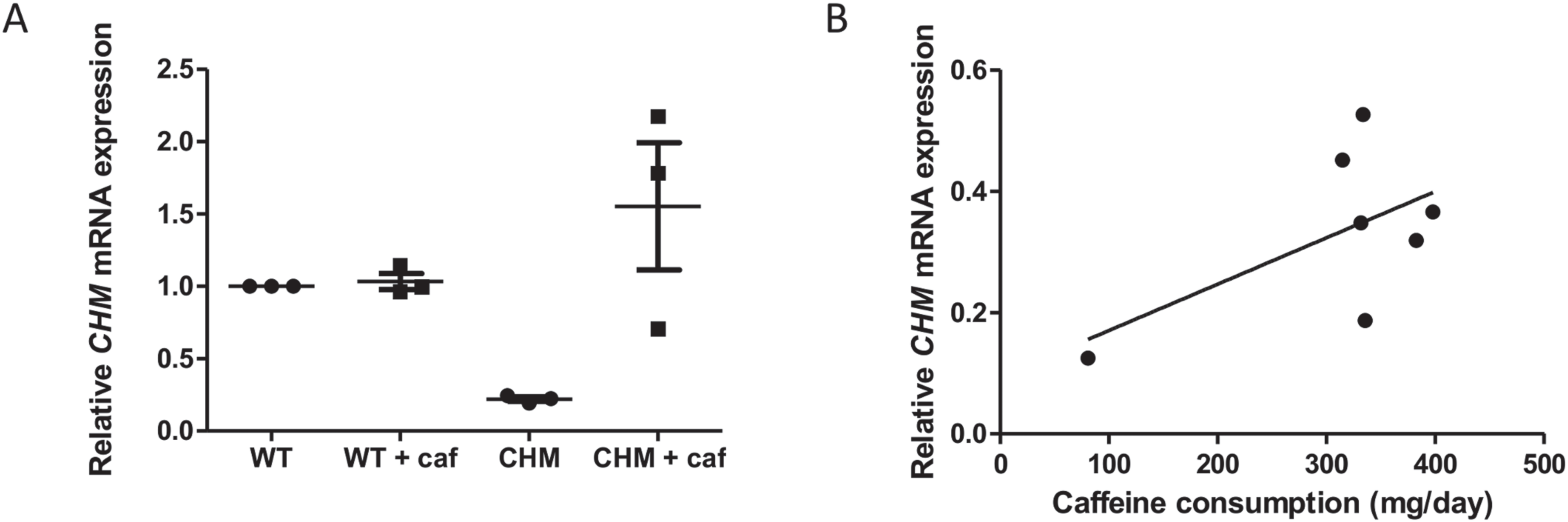
NMD inhibition with caffeine increases *CHM* mRNA expression. **(A)** Effect of caffeine on *CHM* mRNA expression in three unrelated patient fibroblasts [p.(R270^*^), p.(R270^*^) and p.(S190^*^)]. Cells were treated with 10 mm caffeine for 4 h and mRNA expression analysed by RT-qPCR. Caffeine significantly increased *CHM* mRNA expression, compared to untreated cells (*P* < 0.05). Data expressed as mean ± SEM (n = 3). **(B)** Patients were asked to complete a food questionnaire, and the average daily caffeine intake was calculated. Correlation between caffeine consumption and relative *CHM* mRNA expression was analysed by Pearson’s correlation (*r* = 0.58). No significant correlation between patient caffeine consumption and *CHM* mRNA expression (*P* = 0.18) was observed.

The effect of caffeine on *CHM* expression was tested in three independent and unrelated patient fibroblast cell lines, two with a p.(R270^*^) mutation and one with a p.(S190^*^) mutation. *CHM* transcript levels in the two p.(R270^*^) patients were 19.3% ± 3.9% and 24.6% ± 2.3%, and for p.(S190^*^) was 22.3% ± 2.1%. Overall, mean untreated *CHM* expression was 22.1% ± 1.5% (Fig. 4A). Treatment with 10 mm caffeine for 4 h increased *CHM* transcript levels in all cell lines, to a mean 155% ± 44% of wild-type levels, a 7-fold increase (*P* < 0.05; Fig. 4A). This confirms that active NMD is inhibited, leading to rescue of PTC-containing transcripts.

To assess whether caffeine intake influenced whole blood *CHM* mRNA transcript levels, the average daily caffeine consumption from the FFQ was determined for each CHM patient. There was no significant difference in daily caffeine consumption between the CHM group (185.4 ± 28.9 mg/day) and the age-matched controls (177.1 ± 28.9 mg/day). The average caffeine intake for patients used in this study (excluding P1, who does not have an NMD-sensitive *CHM* variant) was 310.8 ± 40 mg/day. There was no sign of correlation between patient caffeine consumption and *CHM* mRNA expression in blood (*r* = 0.58, *P* = 0.18; Fig. 4B).

## Discussion

In this study, we have shown that patients with nonsense mutations in the *CHM* gene have 2.8-fold lower levels of *CHM* transcripts compared to controls, indicating that the transcripts are subject to degradation by NMD, but also a proportion of transcripts are escaping destruction. All patient mutations are positioned at least 55 nucleotides upstream of the final exon–exon junction, and therefore likely substrates of NMD. A wide variation in *CHM* expression was observed amongst patients, which did not correlate with genotype, suggesting other factors may be responsible for NMD efficiency. *UPF1* expression, a key NMD facilitator, was also highly variable with no correlation found with *CHM* transcript levels. Linde *et al.* (17) found NMD efficiency to be variable between different cell types transfected with the same PTC-containing genes, suggesting NMD efficiency to be an inherent characteristic of the cell. However, in this study similar levels of *CHM* transcripts were found between two different tissue-specific cell types in two unrelated patients. For this to be a useful patient screening tool for potential response to nonsense suppression, further validation with a greater number of tissues from more patients would be beneficial. In contrast, corresponding *UPF1* transcript levels did vary between different cell types. This is consistent with the study by Zetoune *et al.* (16), which showed NMD efficiency varies between different murine tissues and does not correlate with *UPF1* expression or expression of any other genes encoding proteins involved in NMD.

Our cohort of patients did not show a correlation between disease severity and mRNA expression, although investigation in a larger patient cohort would increase sensitivity. The role of NMD in the regulation of normal gene expression is becoming more apparent. Investigation in non-disease individuals may be valuable to elucidate the causes of variation in NMD efficiency.

In patient 1, [P1; p.(Y42^*^)], *CHM* mRNA levels are comparable to wild-type levels in all cell types, indicating that this transcript is escaping NMD. As this mutation is present near the start of the coding sequence, the AUG-proximity effect may be in play here. In a study of 10 000 human tumours, Lindeboom *et al.* (18) found that NMD efficiency is significantly reduced in transcripts with PTCs located within the first 200 nucleotides of the start codon. In P1, the PTC is located 126 nucleotides from the start codon. An AUG-proximal PTC transcript can evade NMD and trigger translation re-initiation at a downstream codon. Pereira *et al.* (4) showed the boundary for translation re-initiation in the *β-globin* mRNA is between codons 23 and 25. In the *CHM* transcript, the next AUG is present at codon 149, which is unlikely to trigger translation re-initiation. However, NMD efficiency is still lower in AUG-proximal PTC transcripts, even in the absence of a downstream start codon. An alternative mechanism suggests that the transcript is stabilized by interaction of cytoplasmic poly(A) binding protein 1 (PABPC1) and the termination complex. In the short open reading frame of an AUG-proximal PTC transcript, PABPC1 interacting with eukaryotic initiation factor 4G is bought into close proximity with the termination complex at the PTC, leading to an effective termination event, thereby suppressing NMD (19).

**Table 2:** Fibroblast cell lines used in this study

Age	cDNA change	Amino acid change	Stop introduced	Exon	Coriell ID
43		Healthy control			GM23963
48	c.569 C>G	p.(S190^*^)	UGA	5	GM25421
20	c.808 C>T	p.(R270^*^)	UGA	6	GM25383
61	c.808 C>T	p.(R270^*^)	UGA	6	GM25386
28	c.126 C>G	p.(Y42^*^)	UAG	3	Patient skin biopsy
10	c.772 A>T	p.(K258^*^)	UAA	6	Torriano *et al*. (9)

Cells were obtained from Coriell Institute for Medical Research or cultured from patient skin biopsies.

Linde *et al.* (10) showed in a group of cystic fibrosis patients with the same p.(W1282^*^) mutation, patients had varying levels of baseline *CFTR* transcripts, and those with higher levels responded better to the nonsense suppression drug gentamicin. A number of other studies have shown that response to nonsense suppression drugs is highly variable (10,20,21). We have previously shown that treatment with ataluren restores prenylation activity in *CHM^Y42X^* fibroblasts (8). However, in the study by Torriano *et al.* (9), no significant rescue was observed in *CHM^K258X^* fibroblasts and iPSC-derived RPE, which had lower *CHM* transcript levels (~20%). In preparation for a phase 2 clinical trial with ataluren for CHM, levels of baseline mRNA may provide a prognostic indicator of response to treatment. Patients with lower *CHM* transcript levels may benefit from NMD inhibition to increase baseline levels, allowing for more effective read-through. Treatment with caffeine restored *CHM* mRNA expression to wild-type levels in treated cells. Further clinical studies assessing the direct effect of caffeine on NMD and resultant *CHM* mRNA levels following consumption would provide further evidence of therapeutic benefit. However, caution would be required due to the widespread side effects on the body and interactions with many other pathways, and so potentially local delivery would be more applicable. Keeling *et al.* (14) showed in the mucopolysaccharidosis I-Hurler mouse model, co-administration of the NMD specific inhibitor, NMDI-1, together with gentamicin increased enzyme activity compared to gentamicin alone. Recently, an analogue of NMDI-1 called VG1 has also been developed, using a more efficient process (22). The FDA-approved drug amlexanox has been shown to have a dual function by inhibiting NMD and promoting synthesis of full length protein though nonsense suppression (23).

Together, our results show that NMD efficiencies are highly variable in CHM patients, with no correlation with genotype or phenotype. Levels of transcripts did not vary between tissues; hence, measuring baseline mRNA levels in patients with nonsense mutations, if accessible, may act to guide choice of nonsense suppression therapy with or without NMD inhibitor adjuncts.

## Materials and Methods

### Clinical methods

The study protocol adhered to the tenets of the Declaration of Helsinki and received approval from the NRES Committee London Ethics Committee (REC12/LO/0489) and (REC12/LO/0141). Written, informed consent was obtained from all participants prior to their inclusion in this study.

Clinical data were collected from nine male subjects confirmed to have pathogenic variants in the *CHM* gene, (Table 1) including age, ethnicity and visual acuity. All retinal imaging was collected as part of an on-going natural history study of CHM patients for future gene augmentation therapies tailored to nonsense-mediated disease. Previous work has shown delineation of the central hyper-autofluorescent retinal island area to be the most repeatable metric to measure disease state (24). Images were acquired using short wavelength (488 nm) autofluorescence on the Heidelberg Spectralis confocal scanning laser ophthalmoscope (Heidelberg Engineering, Heidelberg, Germany), and area was measured using the vendor software (Heidelberg Eye Explorer Region Finder Version 2.4.3.0).

### Blood collection and RNA extraction

Whole blood (2.5 ml) was collected from the nine CHM affected male patients and six age- and sex-matched healthy controls (Table 1) in PAXgene Blood RNA tubes (QIAGEN, Manchester, UK). These were incubated at room temperature for 2 h to lyse blood cells. Tubes were then transferred to −20°C until frozen and subsequently stored at −80°C, until further processing. Prior to RNA extraction, tubes were thawed to room temperature. RNA from blood was extracted using the PAXgene Blood RNA kit (QIAGEN), according to the manufacturer’s instructions.

### Fibroblast cell culture and dosing

Three patient and one healthy control fibroblast lines were obtained from Coriell Biorepository or cultured from skin biopsies, as previously described (8; cell line details are listed in Table 2). Cells were maintained in Dulbecco’s Modified Eagles Medium, high glucose, supplemented with 15% FBS and Penicillin/Streptomycin (ThermoFisher Scientific, Paisley, UK). For cell collection, a T75 confluent flask was pelleted at 200*g* for 5 min; the pellet was washed in PBS at 200*g* for 5 min at 4°C twice. All liquid was removed and the pellet snap frozen in an alcohol/dry ice bath and stored at −80°C until further processing. RNA extraction from cells was carried out using RNAeasy Mini Kit (QIAGEN), following the manufacturer’s instructions. For dosing experiments, fibroblasts were plated in 24 well plates at a seeding density of 100 000 cells per well for 48 h. Media were replaced with antibiotic free growth medium, containing 10 mm caffeine (Sigma-Aldrich, Dorset, UK). Cells were incubated at 37°C for 4 h. RNA extraction was carried out using RNAeasy Mini Kit (QIAGEN), following the manufacturer’s instructions. Three independent experiments were performed.

### Fibroblast reprogramming to iPSCs and generation of RPE

Wild-type and *CHM^Y42X^* fibroblasts were reprogrammed to iPSCs, using integration free episomal vectors, and subsequently differentiated into RPE, as previously described (25). RT-PCR of RPE-specific marker genes and immunohistochemistry are shown in supplementary data (Supplementary Material, Fig. S1). RNA extraction was carried out using RNAeasy Mini Kit (QIAGEN), following manufacturer’s instructions. *CHM^K258X^* fibroblasts and iPSC-derived RPE, as well as the corresponding RNA, were generated as previously described (9).

### RT-qPCR

cDNA was synthesized from 500 ng of RNA using the Superscript III First Strand cDNA synthesis kit (Invitrogen), according to the manufacturer’s instructions. Transcript levels were analysed using SYBR Green MasterMix (ThermoFisher) on a StepOne Real-Time PCR system (Applied Biosystems, ThermoFisher, Paisley, UK), under standard cycling conditions. All samples were assayed in triplicate. Primers used for RT-qPCR are listed in Table 3. *GAPDH* was used as a reference gene. As the forward *CHM* primer overlapped the p.(Y42^*^) mutation in P1, the 5′ – CGTCAGACATCAGCAGGAGC (forward) and 5′ – GGATTTGGTGGAGGGGGACA (reverse) primers were used to analyse *CHM* transcript levels in P1 blood, fibroblasts and iPSC-derived RPE.

### Patient caffeine consumption

A total of 25 CHM male patients and 25 age- and sex-matched control subjects were asked to complete a food frequency questionnaire (FFQ) on their average consumption of various foods and drinks over the past 12 months. The validated FFQ comprised a list of 147 food items and participants were asked to indicate their usual consumption from one of nine frequency categories ranging from “never or less than once per month” to “six or more times per day.” (26). Individuals would have been excluded if their answers to >10 items had been left blank, but this was not true for any of the participants. The amount of caffeine in food and drink items was calculated using a database with composition values obtained from the USDA Food Composition Databases (Accessed October 2018). Specifically, using data derived from the USDA National Nutrient Database for Standard Reference Legacy Release (April 2018) and USDA Branded Food Products Database to enable average caffeine consumption (mg/day) to be calculated for each patient.

**Table 3:** RT-qPCR primer sequences

*CHM* forward	5′ - AGAAGCTACTATGGAGGAAAC
*CHM* reverse	5′ – TTCCTGGTATTCCTTTAGCC
*UPF1* forward	5′ – GCTGTCCCAGTATTAAAAGG
*UPF1* reverse	5′ - CAGTGGTGCTTCAGTTTTAG
*GAPDH* forward	5′ - CTTTTGCGTCGCCAG
*GAPDH* reverse	5′ - TTGATGGCAACAATATCCAC

### Statistical analysis

All data are expressed as mean ± SEM. Differences between control and patient groups were analysed by Mann–Whitney test. Relationship between *CHM* and *UPF1* transcript levels were analysed by Pearson’s correlation. To assess the relationship between *CHM* mRNA expression and clinical phenotype, multivariate regression analysis for subject age, FAF area and mRNA level was undertaken (JMP13, Marlow, Buckinghamshire, UK). The effect of caffeine treatment on cells was analysed by Kruskal-Wallis analysis, followed by Dunn’s multiple comparisons test. Correlation between caffeine consumption and *CHM* mRNA levels was analysed by Pearson’s correlation. A *P*-value of <0.05 was considered significant.

## Supplementary Material

Supplementary Data
