# Supplementary material for: Nonsense-mediated mRNA decay efficiency varies in choroideremia providing a target to boost small molecule therapeutics
Source: Hum Mol Genet. 2019 Jan 23;28(11):1865–71. doi: 10.1093/hmg/ddz028 (PMC6522067; doi:10.1093/hmg/ddz028)
Supplement: Supplementary Data [file supplemental_data_hmg_ddz028_supp.pdf]

Supplemental Data

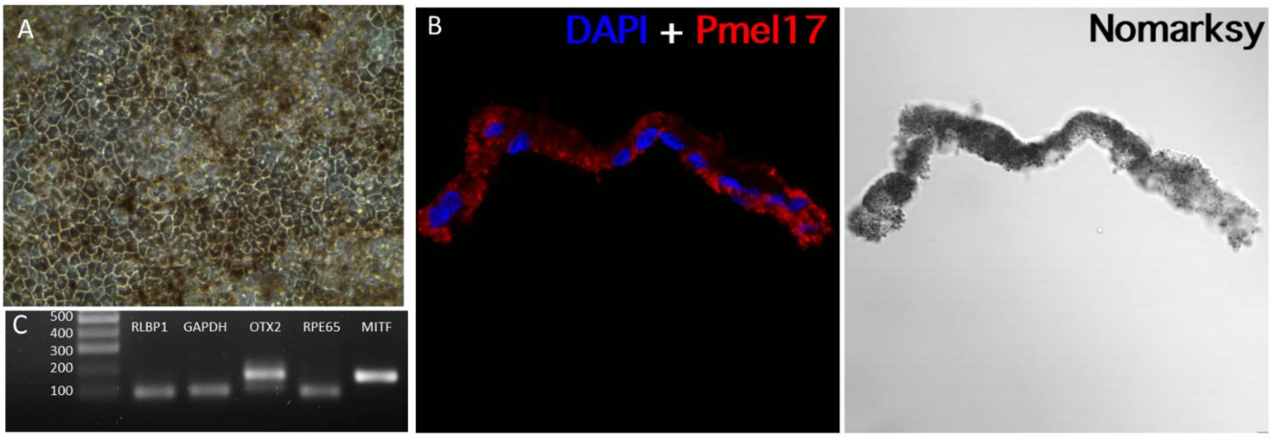

Figure S1: Characterisation of *CHM*^{Y42X} patient derived iPSC-RPE. **(A)** Differentiated iPSC-RPE, displaying characteristic cobblestone morphology. **(B)** Immunostaining of iPSC-RPE sections stained for Pmel17 (red) and counterstained for DAPI (blue). **(C)** RT-PCR gel electrophoresis of RPE cell markers.
